# Historical Trends in Incidence of Breast Cancer in Shanghai, Hong Kong and Los Angeles, 1973–2012: A Joinpoint and Age-Period-Cohort Analysis

**DOI:** 10.3389/ijph.2021.603810

**Published:** 2021-03-17

**Authors:** Hang-Hang Luan, Li-Sha Luo, Zhi-Yan Lu

**Affiliations:** ^1^ Department of Forensic Medicine, Zhongnan Hospital of Wuhan University, Wuhan, China; ^2^ Center for Evidence-Based and Translational Medicine, Zhongnan Hospital of Wuhan University, Wuhan, China

**Keywords:** breast cancer, incidence, historical trends, joinpoint analysis, age-period-cohort analysis

## Abstract

**Objectives:** This study aimed to estimate the long-term trends of breast cancer incidence in Shanghai, Hong Kong, and Los Angeles (LA)**.**

**Methods:** Data were obtained from Cancer Incidence in Five Continents (*CI5plus*) database. The average annual percent change (AAPC) was conducted by joinpoint regression analysis, and the age, period and cohort effects were estimated by age-period-cohort (APC) analysis.

**Results:** The age-standardized incidence rates (ASIRs) in LA were higher than Shanghai and Hong Kong. During 1988–2012, the ASIRs significantly decreased in white women in LA (AAPC = −0.6%, 95% CI: −0.9% to −0.4%) while increased in Shanghai (2.5%: 2.1%–2.9%) and Hong Kong (2.2%: 2.0%–2.5%). The APC analysis revealed significantly increased effects of age and period, and decreased effect of birth cohort.

**Conclusion:** Although age and cohort effects were relatively strong, the period effect may be the key factor affecting trends of incidence, which may be caused by increasing exposures to carcinogens and risk factors. Therefore, more effective measures should be carried out promptly to protect high-risk populations such as elder women, to avoid exposures to risk factors of breast cancer.

## Introduction

Cancer has been recognized the major threat to health globally, which caused a total of 4.11 million deaths in 2017 [1]. Worldwide breast cancer is the most frequently diagnosed cancer and the first leading cause of cancer death among women. There were about 1.67 million new diagnosed breast cancer cases worldwide in 2012, which accounted for 25% of all new cancer cases in women [2]. According to the global burden of disease (GBD) study, countries with high socio-demographic index (SDI) had the higher mortality of breast cancer, while the lowest odds were concentrated in countries with low SDI [3]. With the development of medical technology, the survival rate in breast cancer had increased worldwide, but it varied in different countries [4]. The 5-years survival of breast cancer in China was 73.1%, which was lower than western countries (90.5%) [5]. When the prognosis was not ideal for breast cancer, earlier detection, diagnosis and treatment were the main means to reduce its burden.

There were extensive studies focused on the etiology of breast cancer in recent years, and many factors were proven to be related to its development [6–8]. Established risk factors for breast cancer included gene mutation, advancing age, race, family history, reproductive factors, lifestyles, socioeconomical and environmental factors [8–10]. The patterns and trends in breast cancer incidence have been influenced by early screening, diagnosis and population risk factors, which are not well clarified. Investigation of changing patterns on cancer incidence can provide references for deep exploration of the etiology of the illness [11]. However, previous research on breast cancer had mainly focused on its mortality and risk factors [2], Wang, et al conducted age-period-cohort analysis for breast cancer mortality in four different countries to estimate its trends, and Sun, et al analyzed the risk factors and preventions of breast cancer [9, 12]. At present, there were few studies provided comprehensive analysis for underlying reasons of long-term age-specific incidence trends, especially in different populations in Western and Eastern countries. As we know, the United States and China are the largest and representative developed and developing countries around the world, respectively. In recent decades, China had experienced profound social transformation in terms of economy and sciences and technology [13, 14]. Although the United States had not experienced profound transformation, it had experienced many major social events. Therefore, it is worth making a historical comparison of breast cancer incidence between these two countries. Considering that the different cities in largest countries have substantial disparities in the influencing factors on breast cancer, such as economic development, health systems and environment, and so on, the present study aimed to investigate the historical trends of breast cancer incidences in Shanghai and Hong Kong in China compared with Los Angeles (LA) in the United States to explore the more specific causes of the potential risks, thereby to give hints on resource allocation targeting vulnerable groups for the prevention of breast cancer, as well as provide certain etiologic implications on breast cancer incidence for different cities. We first investigated the historical trends in breast cancer incidence within different cities by joinpoint regression analysis, then analyzed if there were any differences in the net effects of age, period and cohort on incidence by age-period-cohort (APC) analysis with intrinsic estimator (IE) algorithm. Finally, we discussed the underlying reasons for these trends in detail, to identify vulnerable populations and provide certain etiologic implications on the prevention and control of breast cancer.

## Methods

### Data Source

Data were extracted from Cancer Incidence in Five Continents (*CI5plus*) database, which provide annual incidence cases and rates for 28 major cancer types in 124 selected populations from 108 cancer registries, and the incidence accuracy was relied on the local registries [15–17]. There were five registries in China, namely Shanghai, Hong Kong, Jiashan, Zhongshan and Harbin, included in this database. Due to the availability of data for long-term trends, two cities in China, Shanghai and Hong Kong, were included in our study. Los Angeles is the only city that has been listed separately to report cancer incidence in the United States, so we selected it as the comparison in our analysis. These three cities, including Shanghai, Hong Kong and Los Angeles in our study, were all the most economically developed cities in their home countries, and they all had the higher prevalence rates of breast cancer according to the previous studies, so they were representative and comparable to some extent. Additionally, we distinguished the white and black populations in Los Angeles because of the profound racial characteristics in the United States. The annual incidence cases and age-specific rates for each population were ascertained for the period 1973–2012, and the age-standardize incidence rates per 10,000 were calculated according to the world standard population [18, 19]. To better match the APC model, we also obtained the average incidence rates of breast cancer for each consecutive 5-year periods including 1988–1992, 1993–1997, 1998–2002, 2003–2007 and 2008–2012, and 12 five-year age-groups from 30–34 years to 80–84 years. In *CI5plus* database, breast cancer was identified based on the Tenth Revision of the International Classification of Diseases (ICD-10) (code as C50).

### Statistical Analysis

Joinpoint regression analysis was used to estimate the long-term trends of breast cancer incidence among these four populations during 1988–2012 in our study. We assumed the incidence rates of breast cancer followed a Poisson distribution, and identified the time points in which the trends significantly changed according to the Monte Carlo Permutation [20, 21]. The direction and magnitude of changes were presented by the annual percent change (APC) and average annual percent change (AAPC), and the latter means the overall trends during 1988–2012 for each population using the best-fit joinpoint model [20, 22–24]. The APC, AAPC and corresponding 95% confidence interval (CI) for age-standardized and age-specific incidence rates were estimated by the model.

To more accurately describe the accumulated health risks, an age-period-cohort framework was carried out to estimate the independent effects of these three dimensions from the age-specific incidence rates. The APC model was plagued by un-identification problem because of the linear relationship between age, period and cohort (cohort = period-age) [25–27]. Therefore, intrinsic estimator algorithm was conducted to address the collinearity limitation, which was confirmed estimable, valid and unbiased [14, 25, 26, 28]. In addition, the relative risks (RRs) were calculated to the exponential value (exp (coef.) = ecoef.) to more intuitively explain the estimated parameters, which denoted the incidence risk of a particular age, period and cohort relative to each average level.

In our study, the Joinpoint regression analysis was implemented using Joinpoint software from the Surveillance Research Program of the US national Cancer Institute. The APC model was performed using the Stata 12.0 software (Stata Corp, College Station, TX, United States). Results with a 2-sided and *p* value <0.05 were considered statistically significant.

## Results

### The Long-Term Trends of Age-Standardized Incidence Rates of Breast Cancer Among the Populations

**FIGURE 1:**
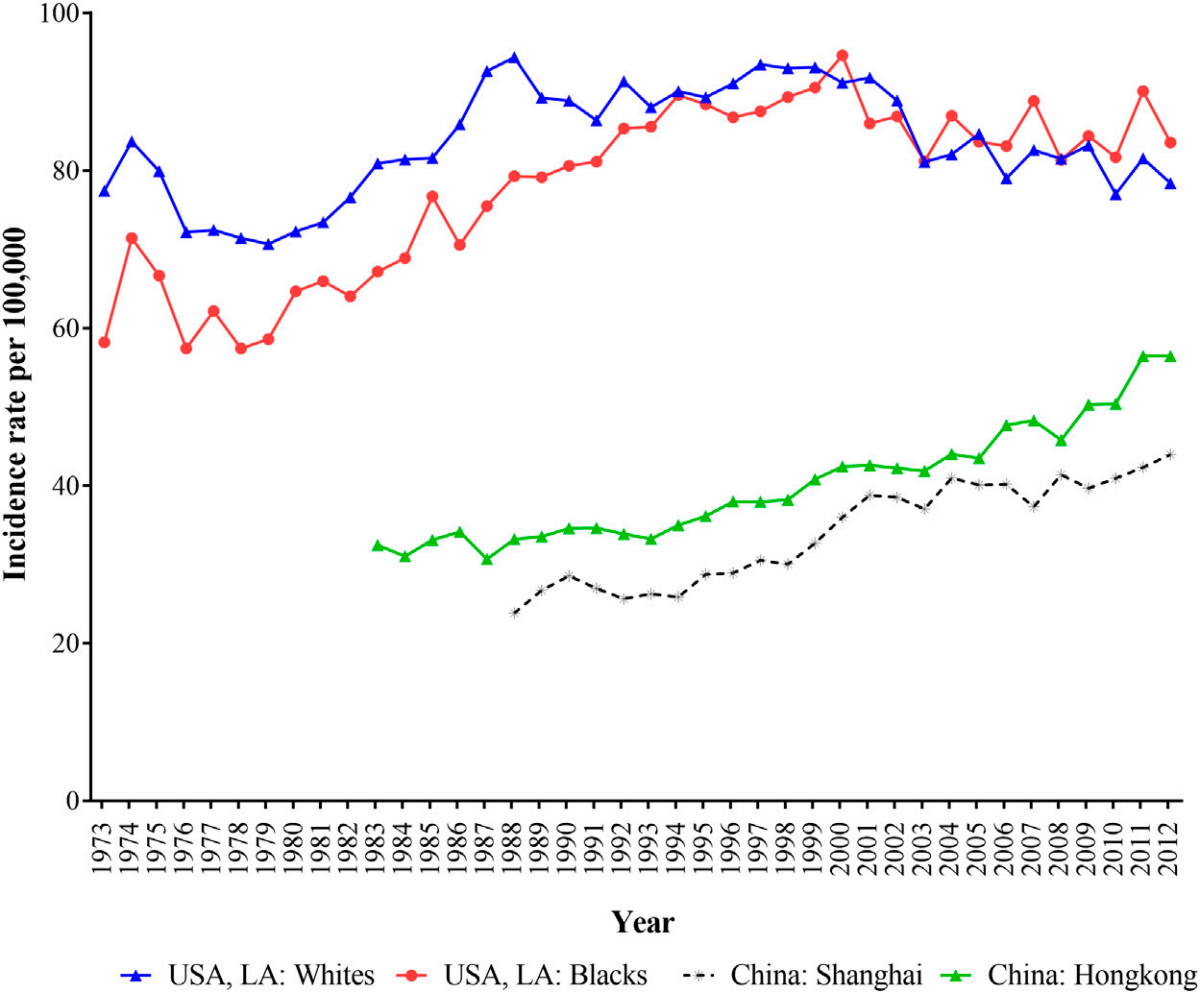
The age-standardized incidence of breast cancer for women in Hong Kong, Shanghai, and whites and blacks in Los Angeles during 1973–2012.

Figure 1 depicted the trends of age-standardized incidence rates (ASIR) of breast cancer in Hong Kong, Shanghai, and whites and blacks in LA during 1973–2012. The ASIRs in LA for both white and black populations were all higher than those in shanghai and Hong Kong, and Shanghai had the lowest breast cancer incidence. In LA, white women had higher incidence of breast cancer than black women before 2003, but after 2003, this trend had reversed. In general, the ASIR of breast cancer for black population in LA, Shanghai and Hong Kong showed upward trends except for white women in LA.

### The Overall and Age-Specific Trends of Breast Cancer Incidence Rates Among the Populations

The APC and AAPC of breast cancer cases and ASIR by four populations from 1988 to 2012 were presented in Table 1. All incidence cases of breast cancer showed upward trends between 1988 and 2012 and the largest increase of cases was found in Hong Kong, which increased by an average of 5.5% per year during the study period. The ASIR for white women in LA decreased by an average of 0.6% per year from 1988 to 2012. Black women in LA showed a decreasing trend during 1995–2012 (APC was −0.4%), but there was no statistical significance in the whole study period. The ASIR for women in Shanghai and Hong Kong increased by 2.5% (2.1–2.9) and 2.2% (2.0–2.5) per year from 1988 to 2012, respectively.

**TABLE 1:** The annual percent change (APC) and average annual percent change (AAPC) of breast cancer cases and age-standardized incidence for women in Hong Kong, Shanghai, and whites and blacks in Los Angeles during 1988–2012.

City	Segments	Cases		ASIR	
United States, LA: White	Trend 1	1988–2001	0.9 (0.4,1.3)*	1988–2000	0.3 (−0.2,0.8)
	Trend 2	2001–2004	−2.1 (−10.2,6.6)	2000–2003	−3.4 (−10.8,4.6)
	Trend 3	2004–2012	0.8 (−0.1,1.7)	2003–2012	−0.6 (−1.3,0.2)
	AAPC (95% CI)	1988–2012	0.3 (0.1,0.5)*	1988–2012	−0.6 (−0.9,−0.4)*
United States, LA: Black	Trend 1	1988–1995	3.2 (1.7,4.7)*	1988–1995	1.9 (0.5,3.4)*
	Trend 2	1995–2012	0.9 (0.6,1.3)*	1995–2012	−0.4 (−0.7,−0.0)*
	AAPC (95% CI)	1988–2012	1.4 (1.1,1.6)*	1988–2012	0.1 (−0.2,0.4)
China, Shanghai	Trend 1	1988–1990	11.6 (−2.1,27.1)	1988–1998	1.8 (0.6,3.0)*
	Trend 2	1990–1994	−1.5 (−7.3,4.7)	1998–2001	8.4 (−5.4,24.1)
	Trend 3	1994–2004	5.6 (4.5,6.6)*	2001–2012	0.9 (0.1,1.8)*
	Trend 4	2004–2012	2.4 (1.4,3.4)*	—	—
	AAPC (95% CI)	1988–2012	3.8 (3.5,4.2)*	1988–2012	2.5 (2.1,2.9)*
China, Hong Kong	Trend 1	1988–2012	5.5 (5.2,5.7)*	1988–2008	2.0 (1.7,2.2)*
	Trend 2	—	—	2008–2012	4.7 (2.0,7.4)*
	AAPC (95% CI)	1988–2012	5.5 (5.2,5.7)*	1988–2012	2.2 (2.0,2.5)*

*p < 0.05.

**FIGURE 2:**
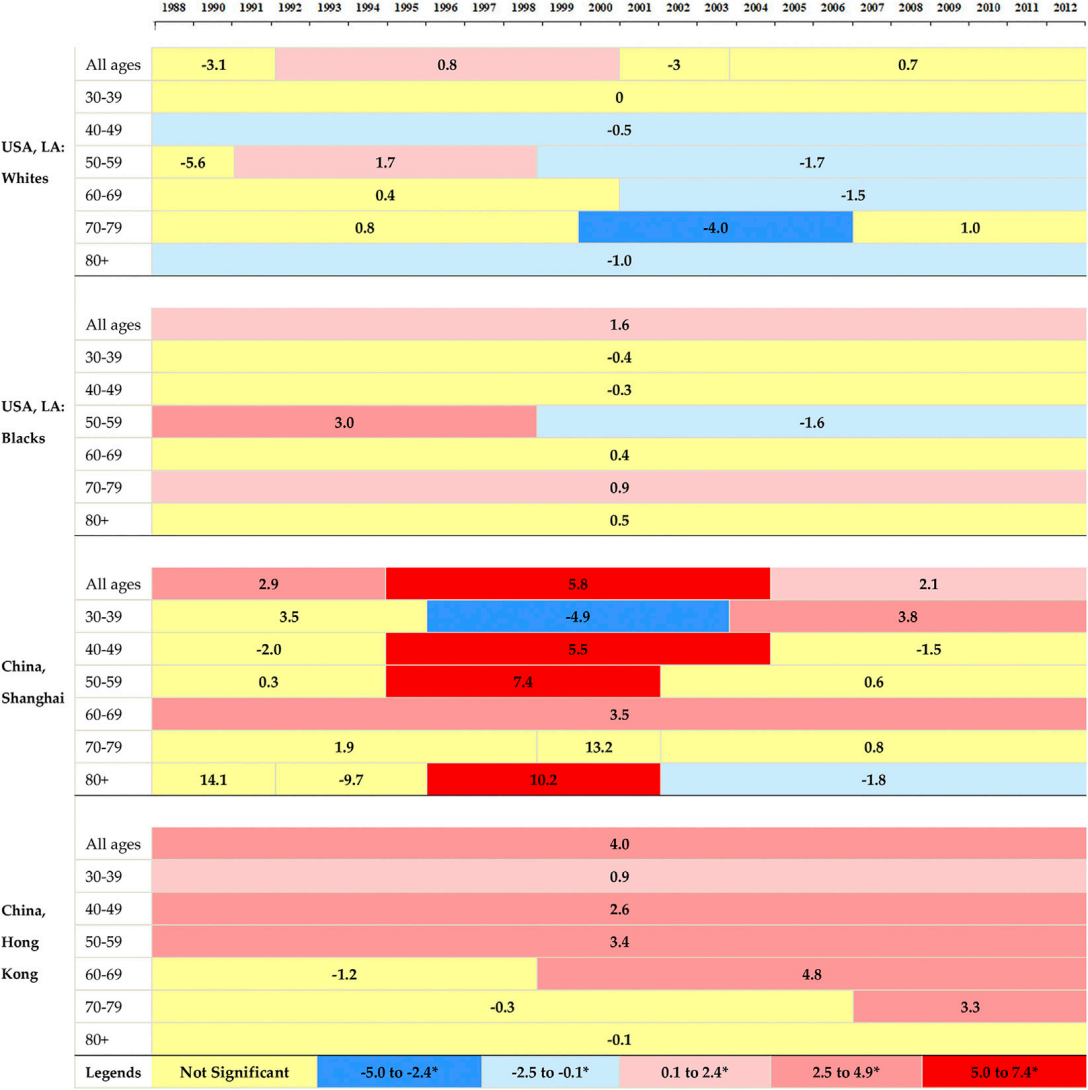
The annual percent changes in overall and age-specific breast cancer incidence by segments of year for women in Hong Kong, Shanghai, and whites and blacks in Los Angeles during 1988–2012.

Figure 2 illustrated the corresponding APCs of the annual age-specific rates of breast cancer. The incidence rates showed decreasing trends in white women in LA for all age groups, and the rates at elder groups decreased more rapidly than those at younger groups. A decreasing trend was observed for black women at the age group 30–49, and followed by an ascending trend after that. The increasing rates in Shanghai were found at the age groups of over 40 years old, with the most rapid increase at age group 70–79. Hong Kong showed upward trends at all age groups and the most rapid increase occurred at group 50–59.

### The Independent Age, Period, Cohort Effects on Breast Cancer Incidence Rates Among the Populations

**FIGURE 3:**
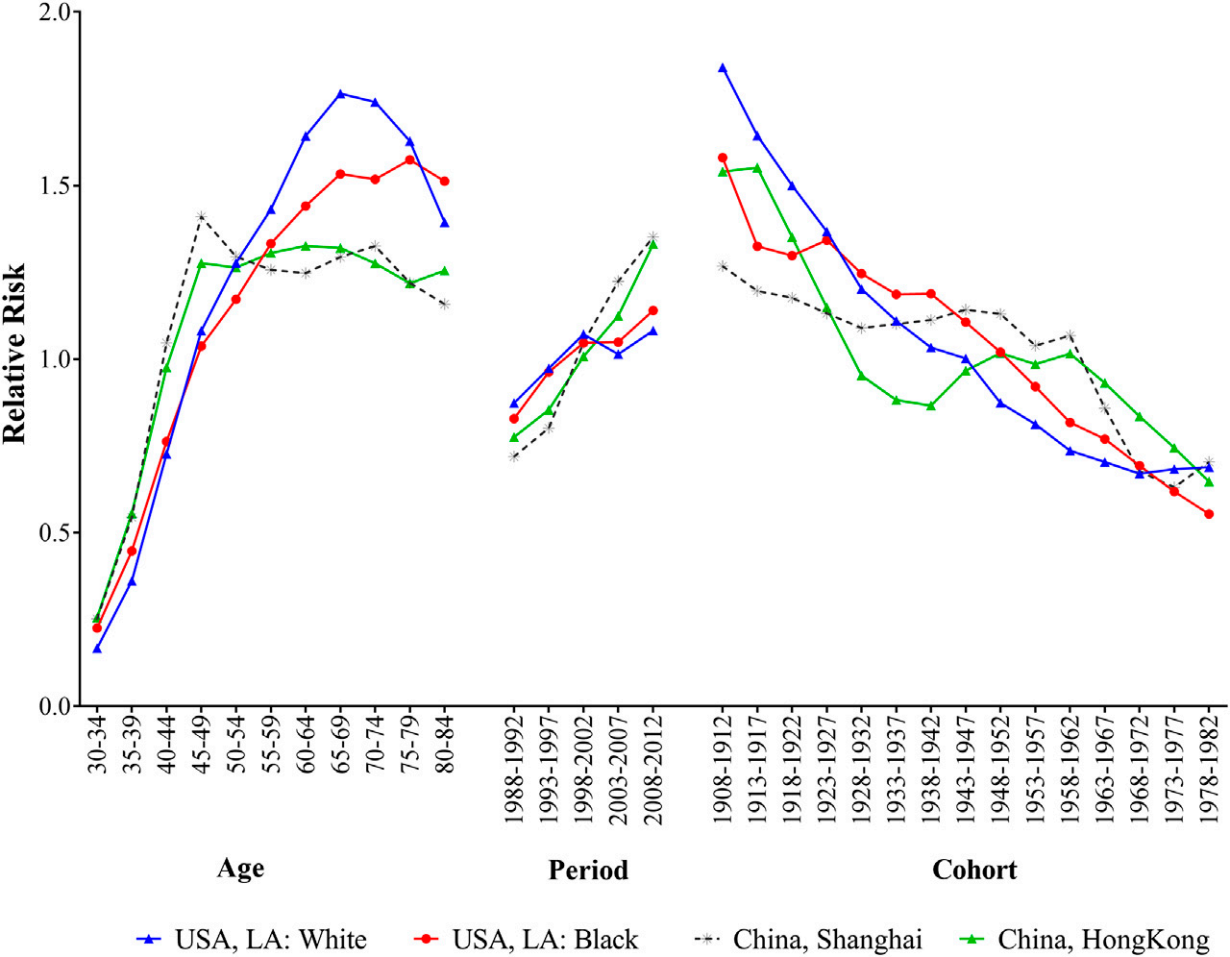
The age, period and cohort effects of breast cancer incidence for women in Hong Kong, Shanghai, and whites and blacks in Los Angeles by age-period-cohort model during 1988–2012.

Figure 3 presented the age, period and cohort effects on breast cancer incidence for four populations. After controlling the effects of period and cohort on breast cancer incidence, the age effects showed that the RRs increased with advancing age among different populations, and the most pronounced differences were observed in elder people. For white population in LA, the RR peaked at the age of 65–69 years, then decreased with advancing age. Black population had a relatively gentle upward trend over 55 years old. The trends of breast cancer incidence rates in Shanghai and Hong Kong had remained relatively stable since the age of 50. The period effects on breast cancer incidence rates showed upward trends for four populations, and the RRs of the incidence increased by 1.24, 1.38, 1.88 and 1.72 times from 1988 to 2012 for white and black population in LA and residents in Shanghai and Hong Kong, respectively. When controlling for age and period effects, the cohort effects on population showed downward trends reduced over generations for all populations, and the disparities were narrowed among people born in 1978–1982.

## Discussion

Based on long-term data from *CI5plus* database, the current study analyzed breast cancer incidence rates across temporal dimensions in Shanghai and Hong Kong compared with white and black populations in LA by Joinpoint regression and age-period-cohort analysis with IE algorithm, which uncovered the underlying causes of long-term trends of breast cancer to estimate when and how each of the three time-dependent parameters affecting these trends [12]. Our discoveries can provide more specific reference for public health policy makers and help them to formulate more targeted and effective prevention and intervention strategies.

Our results revealed that the age-standardized incidence rates presented contrary tendencies between Shanghai, Hong Kong and two populations in LA during the study period. The highest level of incidence risks were observed in white and black populations in LA, while the lowest risk occurred in Shanghai. The disparities between cities in China and USA in our study were consistent with the findings of a mortality trends discovered by Wang et al, which conducted historical trends in breast cancer mortality at country-level in China and United States [12]. More significantly, however, the incidence of breast cancer increased rapidly from 1988 to 2012 in Shanghai and Hong Kong, while decreased or slightly increased in white and black populations in LA. By APC analysis, we found that the incidence trends in these cities can be predominately interpreted by the net period effect, even though age and cohort effects were relatively strong.

Age is an importantly nonmodifiable risk factor for breast cancer, and it was found the breast cancer incidence risk increased with the advancing age in four of the selected populations [29]. Continuously increasing age effects were found in groups of 30–49 years old for four populations, and large disparities were observed after 49 years of age. Shanghai and Hong Kong then maintained relatively stable trends, while white and black populations in LA increased rapidly from 50 to 69 years, which indicated women aged 50–69 years were the highest-risk population for breast cancer in LA, overlapping with menopause in women. Biological factors and aging process might be the main reasons for the increasing effect of breast cancer with age, and elder population might experience more exposure to many high-risk factors for breast cancer [30, 31]. There is a multi-step process for the development of the breast cancer and previous studies have reported that numerous risk factors can increase the possibility of developing breast cancer, including gene mutation, increasing age, family history, lifestyles and so on [6, 9, 32]. One important reason for different patterns of breast cancer incidence was genes, and it was confirmed that BRCA1/2, HER2, Epidermal Growth Factor Receptor (EGFR), and c-Myc have played key roles in the processes of breast cancer [10, 29]. The previous study had identified that there were potential differences in tumor suppressor genes, expression of steroid, growth factor receptors and cell cycle proteins between black and white women, and the incidence of more aggressive subtypes of breast cancer in younger black women had been observed at twice the rate of white women [33]. Besides, the higher incidence risk of breast cancer in postmenopausal women in LA was mainly due to the high prevalence of other postmenopausal disease caused by nutrition, lifestyles and overweight, which was known as “Two-disease model” [12, 34, 35]. For premenopausal women, the exponentially increasing trends of incidence in LA were consistent with Shanghai and Hong Kong, but for postmenopausal women, white and black populations in LA had experienced continuous rises, which had been confirmed in previous studies [12, 34, 36]. The lower age effects for black populations in higher age groups can also be interpreted from barriers to early detection and unequal access to healthcare resources, which had always existed between black and white populations in the United States and would contribute to the different incidence of breast cancer [33]. Therefore, early screening and targeted intervention were essential for postmenopausal women to reduce higher disease burden of breast cancer.

The period effects indicated the immediate effects of the factors on disease incidence and mortality [27]. In the current study, controlling the age and birth cohort effects, continuously increasing period effects were observed in Shanghai and Hong Kong. Compared with 1988, the breast cancer incidence risk increased by 1.88 and 1.72 times for women in Shanghai and Hong Kong in 2012, which indicated that the period effect may be the more crucial factor in the upward trends of breast cancer incidence than the other two effects, and similar trends were also found in the breast cancer mortality in the previous studies [12, 37]. Although there were fluctuations in the two groups of populations in LA, the overall trends were also on the rises. The upward trends of breast cancer incidence might be associated with the changed dietary patterns (increasing fat and caloric intake), higher BMI, and the proper access to screen, diagnosis and management [10]. As far as we know, most female workers in China, especially in the more developed cities like Shanghai and Hong Kong, have access to acquire a free breast ultrasound sponsored by employers every year, and ultrasound has been demonstrated as an effectively diagnostic methods superior to mammography for prevention of breast cancer [36, 38]. Additionally, today’s women are under great pressure from family and career, they lack of time for physical exercise, and higher intake of animal fat, alcohol and tobacco consumption had been confirmed to be positively associated with breast cancer [37]. Benign breast lesions also have a positive relationship with the risk of breast cancer [12, 36]. Overall, an unhealthy lifestyle, increasing detection rate, changes in dietary habits and deterioration of the environment possibly acted in combination to the increase of the breast cancer incidence risk, thus supporting the upward trends of the period effect of breast cancer.

The cohort effects showed declining trends of breast cancer incidence risk for most generations in four selected populations except some cohorts in Shanghai and Hong Kong which level off or slightly increase, which indicated that earlier birth cohort had a higher risk of breast cancer than later cohorts, and similar result was observed in a study conducted by Mubarik S [36]. The possible reasons behind the declining cohort effects were improvement of public health policies and treatment options, and the adequate nutrition intake in childhood for recent birth cohorts, which may reduce the incidence risk of the breast cancer [31, 36, 38]. Additionally, the increase of education level for recent female cohorts may raise their health awareness and socioeconomic status, so breast cancer could be effectively prevented or screened in a timely fashion among later cohorts [27]. Slight increases during 1943–1962 birth cohorts for women in Hong Kong and Shanghai were observed in the current study, which was caused by the combination of the variety of socioeconomic and environmental factors. As we know, the population for 1943–1962 birth cohorts in these two cities were born in the era of the natural disaster and the Great Famine in China, when they can’t get enough foods and nutrition. The childhood nutritional deficiencies and harsh environment at birth had led to the slight increase of breast cancer risk in this generation of women.

Nevertheless, our study had some limitations. Firstly, due to the unavailability of the data, we didn’t analyze the influences of all risk factors on breast cancer, such as dietary changes, physical inactivity and socioeconomic status, and these data would have led to better interpretation of the results. Secondly, we excluded the age groups over 84 years due to the fixed pattern of the IE method. Thirdly, our study included Shanghai, Hong Kong in China and LA, although these three cities were representative cities in China and United States, they can’t fully represent the real conditions of all other cities in their home countries. Therefore, more future studies should be conducted on the breast cancer incidence risk in all cities in China and the United States, to guide the rational allocation of health resources and targeted prevention and intervention strategies. Despite the limitations, our study is beneficial for providing some references to domestic and foreign breast cancer prevention.

In conclusion, the age-standardized incidence of breast cancer in LA for both white and black women were all higher than those in Shanghai and Hong Kong, while upward trends were observed for two latter populations. By APC analysis, the incidence risk of breast cancer increased with advancing age in four selected populations, which was even more pronounced for postmenopausal women in LA. The incidence risk of breast cancer increased with time periods for these four populations. The cohort effects decreased for all selected populations except Shanghai and Hong Kong had slight increase during 1943–1962. Although age and cohort effects were relatively strong, the period effect may be the key factor affecting trends of breast cancer incidence, which may be caused by increasing exposures to carcinogens and high-risk factors. Therefore, more effective measures should be carried out promptly to reduce the burden of breast cancer, especially among high-risk populations such as elder women who are exposure to risk factors of breast cancer.

## Data Availability

The original contributions presented in the study are included in the article/Supplementary Material, further inquiries can be directed to the corresponding author.
